# Comments on “Development and validation of a machine learning-based explainable predictive model for long-term net adverse clinical events in patients with high bleeding risk undergoing percutaneous coronary intervention: results from a prospective cohort study”

**DOI:** 10.1097/JS9.0000000000003137

**Published:** 2025-08-07

**Authors:** Xuesi Chen, Yingchun Bao, Qijun Zhang

**Affiliations:** Department of Cardiology, The Affiliated People’s Hospital of Ningbo University, Ningbo, Zhejiang, China

We read with great interest the recent article by Zhang *et al* entitled “Development and Validation of a Machine Learning-based Explainable Predictive Model for Long-term net Adverse Clinical Events in Patients With High Bleeding Risk Undergoing Percutaneous Coronary Intervention: Results From a Prospective Cohort Study”[1]. This study represents a significant step forward in personalized risk stratification for patients with high bleeding risk (HBR) undergoing percutaneous coronary intervention (PCI). However, several aspects merit further clarification and discussion. The manuscript complies with the TITAN Guidelines 2025—governing declaration and use of AI[2].

The identification of previously overlooked variables such as increased left atrial (LA) diameter, higher levels of β-hydroxybutyrate (β-HB), and anion gap (AG) as strong predictors of net adverse clinical events is particularly insightful. An increased LA diameter reflects cardiac remodeling secondary to chronic pressure or volume overload, and has previously been associated with a higher risk of atrial fibrillation, heart failure, and thromboembolic events[3]. Its selection by the SHAP-based model further supports its prognostic relevance. The role of β-HB is complex. Although it serves as an alternative energy source for stressed myocardium and possesses cardioprotective signaling functions, elevated levels could also indicate metabolic dysregulation[4]. Defining thresholds for prognostic cut-offs and evaluating β-HB in relation to co-existing metabolic disorders warrant further investigation. Similarly, an elevated AG is a readily available marker of systemic metabolic acidosis, often linked to tissue hypoxia or ischemia, which can exacerbate myocardial vulnerability and increase adverse cardiovascular events[5]. Its prognostic value highlights the need for prompt identification and correction of underlying metabolic imbalances.

Translating such models into routine practice, however, faces several challenges. External validation in geographically diverse, multi-ethnic cohorts is essential to confirm generalizability. Data harmonization via common data models, integration into electronic health records and the design of clinician-centered interfaces are crucial to operationalize these tools[6]. Proactive regulatory alignment and bias mitigation strategies will further help ensure equitable and trustworthy model deployment.

To summarize, the study by Zhang *et al* lays a promising foundation for machine learning-driven risk prediction in PCI-HBR patients[1]. Future study should aim to refine mechanistic understanding of novel predictors, validate the model externally, and address practical barriers to clinical implementation.

## Data Availability

All data are included in the article.
